# Insights into Urologic Cancer

**DOI:** 10.3390/cancers15123108

**Published:** 2023-06-08

**Authors:** Niklas Klümper, Jörg Ellinger

**Affiliations:** 1 Department of Urology, University Hospital Bonn, 53127 Bonn, Germany; 2 Institute of Experimental Oncology, University Hospital Bonn, 53127 Bonn, Germany

Collectively, urological malignancies account for a considerable proportion of cancer cases worldwide. Among them, prostate cancer (PCA) is the most frequently diagnosed cancer in men, while bladder cancer (BCA) and renal cancer (RCC) rank among the top 10 most prevalent cancers globally. The high incidence rates pose a significant public health problem. The treatment of urological malignancies often involves complex approaches such as surgery, radiation therapy, chemotherapy, and targeted therapies. However, a better understanding is needed to further enhance the therapeutic management and to improve outcomes for patients. In this editorial, we examine and analyze the key findings from the original articles published in the Special Issue “Insights into Urologic Cancer”. These studies contribute to advancing our understanding of urological malignancies and hold significant implications for patient care and outcomes.

Jirásko et al. [1] explored altered profiles of sulfatides and sphingomyelins in plasma, urine, and tissue samples of patients with RCC. By investigating lipidomic changes, this study enhances our understanding of the molecular mechanisms underlying RCC. The findings highlight the potential of lipid profiling as a diagnostic and prognostic tool. Altered lipid profiles may serve as biomarkers for early detection, monitoring of treatment response, and the identification of novel therapeutic targets. This research will pave the way for personalized approaches and precision medicine in RCC management.

Histopathological discrimination of chromophobe RCC and oncocytoma may be challenging due to a similar appearance. Bin Satter et al. [2] developed an “Chromophobe-Oncocytoma Gene Signature” using a single molecule counting assay and thereby achieved accurate discrimination between chromophobe RCC and oncocytoma. The assay’s ability to provide a reliable and precise distinction is a significant breakthrough and may help to improve classification of these renal tumors.

Metastasis is a major challenge in RCC, often associated with poorer outcomes. Sanders et al. [3] investigated the potential significance of immune cells in the metastatic process. They showed that a higher density of CD103+ cells and a higher ITGAE/CD103 expression were significantly correlated with poor overall survival in clear cell RCC. Understanding the role of tissue resident T-lymphocytes and their correlation with prognosis may lead to the development of novel predictive biomarkers or immunotherapeutic approaches targeting the immune microenvironment in metastatic RCC. This research highlighted the importance of exploring the tumor microenvironment to identify potential prognostic/predictive markers and therapeutic targets.

Tyrosine kinase inhibitors have revolutionized RCC treatment, but resistance remains a challenge. Ding et al. [4] highlighted a potential therapeutic strategy to overcome tyrosine kinase inhibitor resistance and thereby improve patient outcomes. They showed that the circular RNA circDGKD can be targeted to counteract the up-regulation of estrogen receptor β and vasculogenic mimicry in RCC, particularly in response to tyrosine kinase inhibitors. This improved survival in an orthotopic mouse model employing sunitinib treatment.

DJ-1 is involved in various cellular processes and has been implicated in cancer development and progression. Hirano et al. [5] studied DJ-1 expression in the serum of patients with BCA and control subjects. They observed higher DJ-1 levels in BCA patients using a simple ELISA test, indicating that DJ-1 could serve as diagnostic biomarker for BCA. The immunohistochemical detection of DJ-1 in the cytoplasm was associated with poor prognosis.

The study by Gutierrez et al. [6] sheds light on the unique fluorescence patterns associated with urothelial tumor cells. Peri-membrane fluorescence patterns may be assessed to improve urine cytology for early diagnosis and monitoring of bladder cancer. The authors showed that the plasma membrane plays a major role in the maintenance of peri-membrane fluorescence and that stress decreases peri-membrane.

The introduction of the antibody–drug conjugates enfortumab vedotin targeting Nectin-4 revolutionized the treatment of metastatic BCA. However, BCA exhibits diverse histological subtypes with varying prognoses, and the prevalence of Nectin-4 expression remained unclear. Rodler et al. [7] focused on the expression of Nectin-4 in variant histologies of BCA and its prognostic significance. They demonstrated that Nectin-4 expression is weak in sarcomatoid urothelial carcinoma, thereby probably limiting Nectin-4-directed antibody–drug conjugates in patients suffering from this specific subtype.

CD155 is mainly expressed in various cancer cells. Mori et al. [8] studied the expression of CD155 on the membrane and the cytoplasm of urothelial cells. They confirmed a lack of CD155 in normal urothelial cells, whereas CD155 was identified in the cytoplasm and membrane of tumor cells. Membranous CD155 was associated with a shortened recurrence-free survival and cancer-specific survival following radical cystectomy for BCA. A high CD155 expression on tumor cells may lead to tumor immune tolerance and may be targeted by treatment with an anti-TIGIT antibody.

Gemcitabine is a commonly used chemotherapy drug, but resistance often limits its effectiveness. Wang et al. [9] investigated the role of NXPH4 in BCA. They showed that NXPH4 plays a crucial role in BCA progression. NXPH4 contributes to the proliferation, migration, and invasion of BCA by maintaining the stability of NDUFA4L2 and activating reactive oxygen species and glycolysis, thus uncovering the mechanism by which NXPH4 enhances reactive oxygen species production and activates glycolysis through the modulation of NDUFA4L2. This together modulates gemcitabine resistance.

RNA-binding proteins play an essential role in post-transcriptional gene regulation, and their dysregulation has been implicated in cancer. Gu et al. [10] developed an RNA-binding protein risk score based on six genes (AHNAK, MAP1B, P4HB, FASN, LAMA2, and GSDMB) that was an independent predictor of overall survival and could be used for the development of a nomogram in patients with BCA. AHNAK was functionally validated, as were the oncogenic role (proliferation, invasion, and migration) and effect on immune cell infiltration.

Ferroptosis, a form of regulated cell death, has emerged as a potential therapeutic target in various cancers. Zhang et al. [11] explored survival and therapeutic response-related ferroptosis regulators in bladder cancer through data mining and experimental validation. Ferroptosis regulators impact BCA microenvironment and influence BCA survival. A ferroptosis gene signature predicts the effect of chemo- and immunotherapy in BCA. Thus, this research may pave the way for the development of novel therapeutic strategies targeting this cell death pathway.

Immunotherapy has shown promise in the treatment of BCA, but response rates can vary. Shimizu et al. [12] investigated, in a multicenter retrospective analysis, the outcome of patients with urothelial cancer undergoing pembrolizumab therapy. They showed that bone metastases responded only infrequently to pembrolizumab. Furthermore, responses were prolonged in locally unresectable cancers and lymph node metastasis compared with lung and liver metastases. This study highlights the need for personalized treatment strategies based on individual patient characteristics.

Radical prostatectomy is a common treatment option for localized prostate cancer, and it is crucial to consider not only the surgical procedure itself but also the supportive measures provided to patients. Wolf et al. [13] aimed to identify gaps between patient expectations and the actual provision of supportive care in certified and non-certified PCA cancer centers. Interestingly, patients rated the availability of most measures similar in certified and non-certified PCA centers. with no statistically significant difference observed concerning the supportive measures rated most relevant by the patients.

In conclusion, the collection of articles in the Special Issue “Insights into Urologic Cancer” has made substantial contributions to our comprehension of urological malignancies. These studies have shed light on various aspects of urological cancers. The studies have also shed light on emerging therapeutic approaches, such as targeting specific molecular pathways or exploring the role of circular RNAs in overcoming drug resistance.

Looking ahead, the insights gained from these studies open up new avenues for future functional, translational, and clinical research. Further investigations can build upon the knowledge obtained in this Special Issue to refine diagnostic methods, optimize treatment protocols, and develop novel therapeutic interventions. By addressing the gaps in our understanding of urological malignancies, future research endeavors hold the potential to improve patient outcomes and to ultimately contribute to the global fight against urologic cancers.
